# Endocannabinoid contributions to the perception of socially relevant, affective touch in humans

**DOI:** 10.1038/s41386-025-02053-y

**Published:** 2025-01-22

**Authors:** Madeleine R. Jones, Connor J. Haggarty, Gavin N. Petrie, Abigail R. Lunge, India Morrison, Matthew N. Hill, Markus Heilig, Leah M. Mayo

**Affiliations:** 1 Department of Biomedical and Clinical Sciences, Center for Social and Affective Neuroscience, Linköping University, Linköping, Sweden; 2 Department of Psychiatry, Cumming School of Medicine, University of Calgary, Calgary, AB Canada; 3 Hotchkiss Brain Institute, University of Calgary, Calgary, AB Canada; 4 Mathison Centre for Mental Health Research and Education, University of Calgary, Calgary, AB Canada; 5 Department of Cell Biology and Anatomy, University of Calgary, Calgary, AB Canada

**Keywords:** Social neuroscience, Emotion

## Abstract

Social relationships are central to well-being. A subgroup of afferent nerve fibers, C-tactile (CT) afferents, are primed to respond to affective, socially relevant touch and may mitigate the effects of stress. The endocannabinoid ligand anandamide (AEA) modulates both social reward and stress. We thus hypothesized that AEA levels would be associated with the perceived pleasantness of affective touch in humans. Across two studies, we explored perceptions of affective, socially relevant touch and general affective stimuli. In study 1, adult participants (N = 101) were recruited based on presence (CM+) or absence (CM−) of documented childhood maltreatment (N = 52 CM+; N = 49 CM−). In study 2, healthy individuals were randomized to receive an inhibitor of fatty acid amide hydrolase (FAAH; PF-04457845) to increase AEA levels (n = 16) or placebo (n = 29). Outcomes included self-report ratings of touch pleasantness and intensity, valence and arousal ratings of affective images, and plasma levels of endocannabinoids AEA and 2-AG, cortisol, and oxytocin. In study 1, higher AEA levels were associated with a reduced preference for affective, CT-optimal touch. In study 2, pharmacological elevation of AEA resulted in reduced preference for affective touch. These effects were specific to social processing, as AEA levels were not related to ratings of affective images. In contrast to our hypothesis, elevated AEA was associated with reduced pleasantness ratings of CT-optimal, affective touch. This provides novel, in-human data linking AEA to social processing, adding nuance to the rationale for its use as a potential novel therapeutic target in disordered in social processing.

## Introduction

Touch is a vital component of social attachment in mammals [1, 2]. In humans, forms of gentle touch often occur during close affiliative interactions and carry positive affective weight [3]. This likely involves contributions from specialized afferent nerves called C-tactile (CT) fibers (Löken et al. [4], etc). CT afferents convey touch signals to key brain regions, such as posterior insula, which also respond to the mere observation of gentle, affective touch in others [5]. Both experienced and observed affective touch can similarly impact psychophysiological measures of emotion processing [6, 7]. Affective touch can also mitigate feelings of social exclusion [8], and may play a role in stress regulation. For example, heart rate and skin conductance, often used as indicators of stress arousal, are attenuated during touch from a familiar human partner [9–11]. Some studies have begun to explore potential neuromodulatory systems subserving touch processing, including opioids [12] and oxytocin [13]. However, to date, few studies have explored how endocannabinoid (eCB) function may contribute to touch processing in humans.

The eCB system is believed to play a key role in modulating both stress reactivity and social behavior [14–16]. This system is comprised of the endogenous ligands N-arachidonoylethanolamine (anadamide; AEA) and 2-arachidonoylglycerol (2-AG), which bind to the CB1 receptor, mostly expressed in brain, and the CB2 receptor, primarily expressed in the body. Of the eCB ligands, potentiation of AEA activity in particular has garnered significant interest as a potential novel therapeutic mechanism via inhibition of its main degradative enzyme fatty acid amide hydrolase (FAAH). Clinical trials exploring the impact of FAAH inhibition have been completed or are ongoing for a range of disorders characterized by dysregulation of stress and social processing, including social anxiety disorder [17], post-traumatic stress disorder (EudraCT 2020-001965-36), and autism spectrum disorder [18]. Evidence from healthy humans supports the link between AEA and stress reactivity, as elevated AEA can attenuate stress reactivity and promote regulation of negative emotion [19, 20]. However, there is a paucity of information on the role of AEA in social processing in humans, particularly in non-clinical populations.

Here, we aimed to determine whether the eCB system is involved in the perception of socially relevant, affective touch, as well as whether these effects are specific to social stimuli or also evident more broadly in affective processing. In the first study, we assessed how individual variation in the eCB system impacted the perception of CT-optimal affective touch in a heterogenous participant sample with or without documented histories of childhood maltreatment (CM) [21]. We then aimed to confirm our findings in a second study in which AEA levels were pharmacologically elevated with a FAAH inhibitor (PF-04457845; [20]) in healthy adults who completed the same affective touch and affective image tasks.

## Methods

### Study 1 methods

#### Overview

The study was approved by the Regional Ethics Review Board in Linköping, Sweden (Dnr 2015/256-31). Participants were recruited between March 2017 and July 2020 for a study assessing the relationship between documented childhood maltreatment and substance use disorder histories on affective processing, with the primary results published previously [21]. Extensive details regarding the population and other experimental procedures can be found in [21] and Supplemental Materials.

#### Participants

Demographic information can be found in Table 1. In brief, patients were recruited based on documented history of childhood maltreatment (CM). The N = 101 population consisted of n = 52 CM+ and n = 49 CM− matched on other demographic factors. In addition to the prospective CM assessment obtained via electronic medical records, all participants also completed the Childhood Trauma Questionnaire [22].

**Table 1:** Participant characteristics.

*Study 1*				
	Childhood maltreatment (N = 52)	No childhood maltreatment (N = 49)	Total (N = 102)	p-value
Sex: Female	37 (63.8)	25 (51.0)	62 (60.8)	*p* = 0.10
Age	29.1 (3.6)	27.9 (4.3)	28.6 (4.0)	*p* = 0.06
CPRS (Psychiatric symptoms)				
Depression scores (MADRS)	5.9 (4.7)	3.7 (3.7)	4.9 (4.4)	*p* = **0.01**
Anxiety scores	6.9 (4.4)	5.1 (3.7)	6.1 (4.2)	*p* = **0.03**
CPRS total scores	12.3 (8.4)	8.3 (7.1)	10.5 (8.0)	*p* = **0.01**
DERS (Emotion regulation)	38.5 (15.7)	33.4 (13.9)	36.2 (15.1)	*p* = 0.08
AUDIT	6.2 (4.9)	5.1 (5.1)	5.7 (5.0)	*p* = 0.27
DUDIT	2.0 (5.4)	3.1 (6.4)	2.5 (5.9)	*p* = 0.31
CTQ (Childhood trauma)				
Physical abuse	9.1 (4.5)	5.9 (2.2)	7.6 (4.0)	*p* < **0.001**
Sexual abuse	9.6 (6.6)	5.1 (0.5)	7.5 (5.4)	*p* < **0.001**
Emotional abuse	11.8 (5.6)	8.2 (4.6)	10.2 (5.5)	*p* < **0.001**
Physical neglect	8.8 (4.0)	6.9 (3.6)	7.9 (3.9)	*p* = **0.014**
Emotional neglect	12.5 (5.4)	9.5 (5.0)	11.1 (5.4)	*p* = **0.004**
*Total score*	51.8 (20.8)	35.6 (13.7)	44.4 (19.6)	*p* < **0.001**

*Study 2*				
	PBO (N = 29)	FAAHi (N = 16)	Total (N = 45)	p-value
Sex: Female	17 (58%)	9 (56%)	26 (58%)	*p* = 0.88
Age	26.1 (8.4)	24.6 (56%)	25.5 (7.2)	*p* = 0.51
STAI (Anxiety)				
STAI-State	31.1 (6.2)	31.2 (7.8)	31.1 (6.7)	*p* = 0.96
STAI-Trait	35.9 (4.9)	34.6 (5.9)	35.4 (5.3)	*p* = 0.46
AUDIT	4.0 (2.1)	5.3 (2.6)	4.6 (2.4)	*p* = 0.10
DUDIT	0.2 (0.8)	0.1 (0.5)	0.2 (0.8)	*p* = 0.60
PANAS				
Positive	27.4 (5.4)	27.2 (6.9)	27.3 (5.9)	*p* = 0.90
Negative	14.2 (4.3)	16.1 (6.2)	14.9 (5.1)	*p* = 0.22

Data are presented as mean (standard deviation) for continuous measures and N (%) for categorical measures.

*CTQ* childhood trauma questionnaire, *CPRS* comprehensive psychiatry rating scale, *MADRS* montgomery-asberg depression rating scale, *DERS* difficulties in emotion regulating scale, *AUDIT* alcohol use disorders identification test, *DUDIT* drug use disorders identification test, *CTQ* childhood trauma questionnaire, *PANAS* positive and negative affect schedule, *POMS* profile of mood states. Bolded values *p* < 0.05.

#### Behavioral tasks

Behavioral tasks were completed in a fixed order, with the *Observed Touch task* completed first, and the *Affective Image task* completed after a 15 min break. This order was necessary due to the subsequent tasks, which included a stress task, to avoid carry-over effects of stress. The other tasks are reported elsewhere [21].

##### Observed touch task [6, 7]

Participants viewed 6 s videos depicting a left arm resting on a black background, being stroked by a hand at a rate of 3 cm/s (“slow” or CT-optimal) or 30 cm/s (“fast” or CT-non-optimal). Duration of videos was kept constant such that videos of 3 cm/s touch included two strokes, while videos of 30 cm/s included six strokes, producing videos of equal length (6 s). After each video, participants provided ratings of perceived pleasantness (−10 to +10) and intensity (−10 to +10) of the touch. The task took ~12 min to complete. While the study herein only employed Observed touch, neural and psychophysiological responses to Observed touch and Experienced touch are highly related [5, 6].

##### Affective image task [19, 23]

Positive, neutral, and negative images were selected from the International Affective Picture System based on normative ratings of valence and arousal. The task consisted of 12 images per stimulus type (positive, neutral, negative). Participants viewed a single image for 6 s and then rated valence from −4 to +4 and arousal from 0 to 9. The task took ~15 min to complete.

#### Biological samples

Blood samples were collected before and after each task. Both tasks reported here (*Observed Touch Task* and *Affective Image Task*), were completed prior to administration of other tasks (e.g., stress, fear conditioning), with primary results published elsewhere [21]. We calculated an AUC for the relevant biological variables (AEA, 2AG, cortisol). The eCBs AEA and 2-AG were extracted and analyzed using liquid chromatography tandem mass spectrometry (LC-MS/MS), as previously published and described in [19, 20]. Endocannabinoid values were log-transformed due to nonnormality of the distribution; these transformed values were used in all subsequent analyses. Plasma cortisol levels were obtained using the DetectX Cortisol Enzyme Immunoassay kit (Arbor Assays, Ann Arbor, MI, USA) according to manufacturer instructions.

#### Statistical analysis

Analyses of touch perception were carried out using repeated measures analysis of variance (RM-ANOVA) with Touch Type (CT-optimal/slow vs CT-non-optimal/fast) as a within subject factor for ratings of Intensity or Pleasantness, individually. Responses to affective images were analyzed using RM-ANOVA with Image Type (Positive, Neutral, Negative) as a within-subjects factor. Group (CM+, CM−) was entered as a between-subjects factor and AEA and 2-AG levels were entered separately as covariates. However, Group (CM+, CM−) was not a significant factor in any of the analyses and so was dropped from the model to achieve parsimony. Significant effects were followed up with Tukey’s post hoc comparisons.

To further explore the relationships between touch preference and biological measures, we created differences scores, as previously described [6]. Specifically, CT preference was calculated as [score for CT-optimal touch] – [score for non-optimal touch]. Analyses were carried out using SPSS and graphed using GraphPad Prism.

### Study 2 methods

#### Overview

This double-blind, placebo-controlled study was approved by the Linköping Regional Ethics Review Board and the Swedish Medical Products Agency (EudraCT 2016-005013-47). The current data is an unpublished subset of a larger experimental medicine trial with primary outcomes reported previously [20]. Briefly, qualifying participants were randomized to receive the FAAH inhibitor PF-04457845 4 mg/day or placebo for 10 days. On days 9 and 10 of dosing, participants completed the tasks described herein, in addition to those described elsewhere [20]. Critically, the tasks described below (*Observed touch task, Affective image task*) always followed the non-stress control task (see Supplemental Materials for more information). Behavioral tasks were carried out as described in Study 1 “Methods”.

#### Participants

Participants over 18 years old were recruited through flyers and online advertisements from July 2017 to May 2018. Eligibility was confirmed at a screening session, followed by informed consent, and then medication distribution. Sessions were completed on consecutive days (days 9 and 10 of dosing) at 12.00 or later. Though 30 participants per treatment group were originally included, a pharmacy error resulted in only n = 16 receiving active drug as confirmed by plasma drug levels. Critically, those individuals are indistinguishable from the placebo-allocated group [20]. Demographics are in Table 1.

#### Drug intervention

PF-04457845 is a highly selective, orally available irreversible inhibitor of FAAH originally developed for pain and insomnia. PF- 04457845 tablets (4 mg) and visually indistinguishable placebo were provided by Pfizer (Groton, VT). Blinding and randomization were carried out by a contractor (Oriola, Stockholm, Sweden) insulated from the investigators. Labeled medication boxes were delivered to the Linköping University Hospital pharmacy. Adherence was confirmed by analysis of PF-04457845 in plasma at study completion. Participants and study personnel were blinded to drug administration until study completion.

#### Procedures

Blood samples were collected at T −15, T0, T+15, T+30, T+45 relative to administration of a stress or control task, with stress and control tasks completed on separate days (see Supplemental Materials for more details). Data presented here all came from the control task day, as the relevant tasks (*Observed Touch Task* and *Affective Image Task*, described below) were always completed post-control task. Biological samples were collected and analyzed in an identical manner as Study 1, with the addition of oxytocin analysis, as described [13].

#### Behavioral tasks

Behavioral tasks (*Observed Touch Task* and *Affective Image Task*) were identical to Study 1. The *Observed Touch Task* was always completed last.

#### Statistical analysis

All analyses were performed as described for Study 1, with the exception that treatment (FAAHi or PBO) was entered as a between-subjects factor. AEA was not entered as a co-variate in this analysis, as AEA levels were significantly different between groups.

## Results

### Study 1 results—individual variation in AEA levels

#### Demographics

The average age was 28.4 ± 4.0 years, and 56% (n = 57) was female. Demographic factors did not differ across groups.

#### Biological samples

**Fig. 1:**
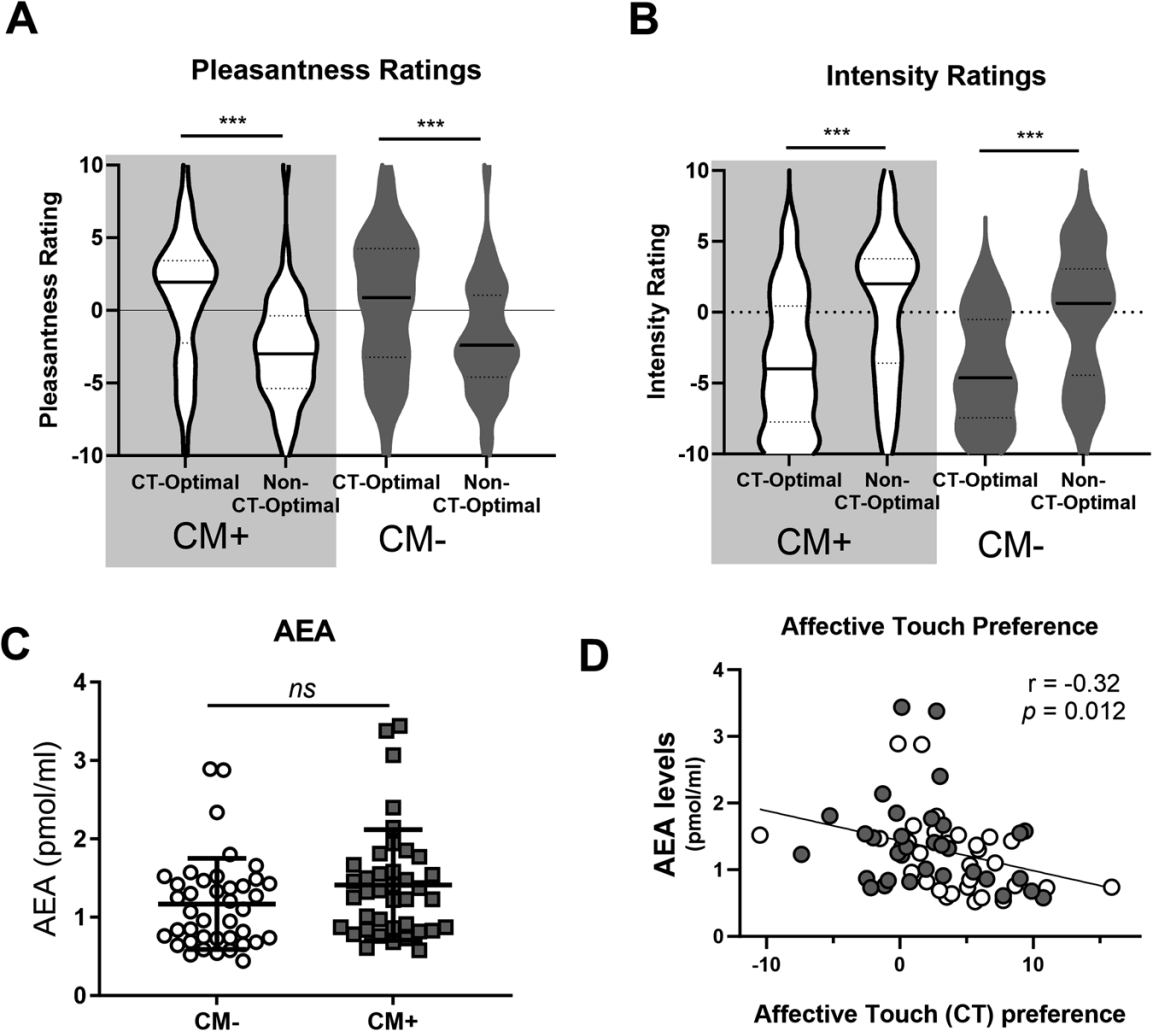
Preference for affective, CT-optimal touch is inversely related to anandamide levels. All participants rated CT-optimal touch as more pleasant (**A**) and less intense (**B**), but these ratings did not differ between CM+ and CM− Groups (**A**, **B**). There were no significant differences in basal AEA levels between CM+ and CM− Groups (**C**). Across all participants, higher AEA levels were associated with reduced preference for socially relevant, affective touch, calculated as [(pleasantness of CT-optimal touch) – (pleasantness of CT-non-optimal touch)] (**D**). *** p < 0.001.

**Fig. 2:**
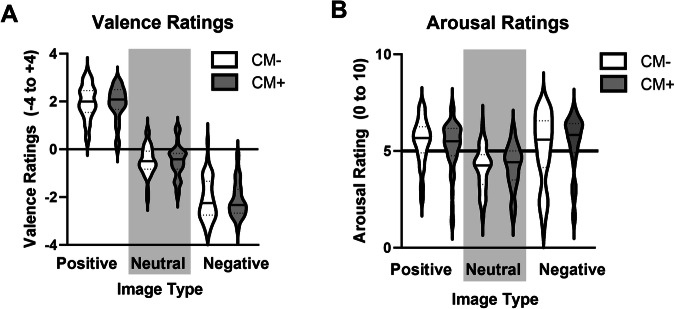
Ratings of affective images are not impacted by CM status or AEA levels. All participants rating positive images as more positive than neutral, and negative as more negative than neutral (**A**). Positive and negative images were rated as more arousing than neutral (**B**). There was no effect of CM status (CM+, CM−) or AEA levels on responses to affective images.

There were no significant group differences in AEA (p = 0.10; Figs. 1C), 2-AG (p = 0.66), or cortisol (p = 0.54) between Groups (CM+, CM−). Oxytocin levels were not measured in Study 1.

#### Observed touch task

##### Pleasantness ratings

There was a main effect of Touch Type (F(1,63) = 23.40, p < 0,001, partial η^2^ = 0.27; Fig. 1A), a significant interaction between Touch Type and AEA levels (F(1,63) = 7.13, p = 0.01, partial η^2^ = 0.10) but no between-subjects effect of AEA (p = 0.54). Post-hoc tests revealed that, as expected, slow, CT-optimal touch was rated as significantly more pleasant as fast, non-optimal touch (t(82) = 6.64, p < 0.001). This effect was not impacted by CM type as indicated on the Childhood Trauma Questionnaire (CTQ; emotional abuse, physical abuse, sexual abuse, emotional neglect, or physical neglect; p’s = 0.21 – 0.77).

To explore the relationship between touch perception and AEA levels, we calculated CT-Touch Preference as done previously [6]. We found a significant correlation between AEA levels and preference for CT-optimal touch (r(63) = 0.32, p = 0.012; Fig. 1D), such that those with *lower* AEA had *greater* preference for CT-optimal touch. There was no relationship between Touch Preference and 2-AG (p = 0.32) or Cortisol (p = 0.39), nor was there a correlation between Intensity Preference scores and any biological measure (all p > 0.05).

##### Intensity ratings

There was a main effect of Touch Type (F(1,68) = 14.1, p < 0.001, partial η2 = 0.17; Fig. 1B) on intensity ratings. Post-hoc testing revealed that slow, CT-optimal touch as significantly less intense than fast, non-optimal touch (t(87) = 6.209, p < 0.001). There was no significant interaction between Touch Type and AEA (p = 0.18), nor was there a between-subjects effect of AEA (p = 0.39).

#### Affective picture task

##### Valence ratings

There was a main effect of Image Type (F(2,154) = 172, p < 0.001, partial η^2^ = 0.69; Fig. 2A), but no effect of AEA (p = 0.22) or Image Type × AEA interaction (p = 0.66). Post hoc follow-up tests revealed that Positive pictures were rated more positively than Neutral (t(1,99) = 28.6, p < 0.001) and Negative (t(1,99) = 37.8, p < 0.001) images, while Neutral images were rated more positive than Negative images (t(1,99) = 23.2, p < 0.001).

##### Arousal ratings

There was a main effect of Image Type (F(2,154) = 9.14, p < 0.001, partial η^2^ = 0.11; Fig. 2B) but no significant effect of AEA (p = 0.63) or AEA x Image Type interaction (p = 0.83). Ratings of Arousal were greater for Positive (t(1,98) = 10.8, p < 0.001) and Negative (t(1,98) = -9.80, p < 0.001) images compared to Neutral, but were not significantly different from each other (p = 0.22).

### Study 2 results—pharmacological elevation of AEA

#### Demographics

The average age was 25 ± 7.7 years old and 45% of the sample was female. Groups (PBO, FAAHi) did not differ in age, gender, or any other demographic factor.

#### Biochemical measures

**Fig. 3:**
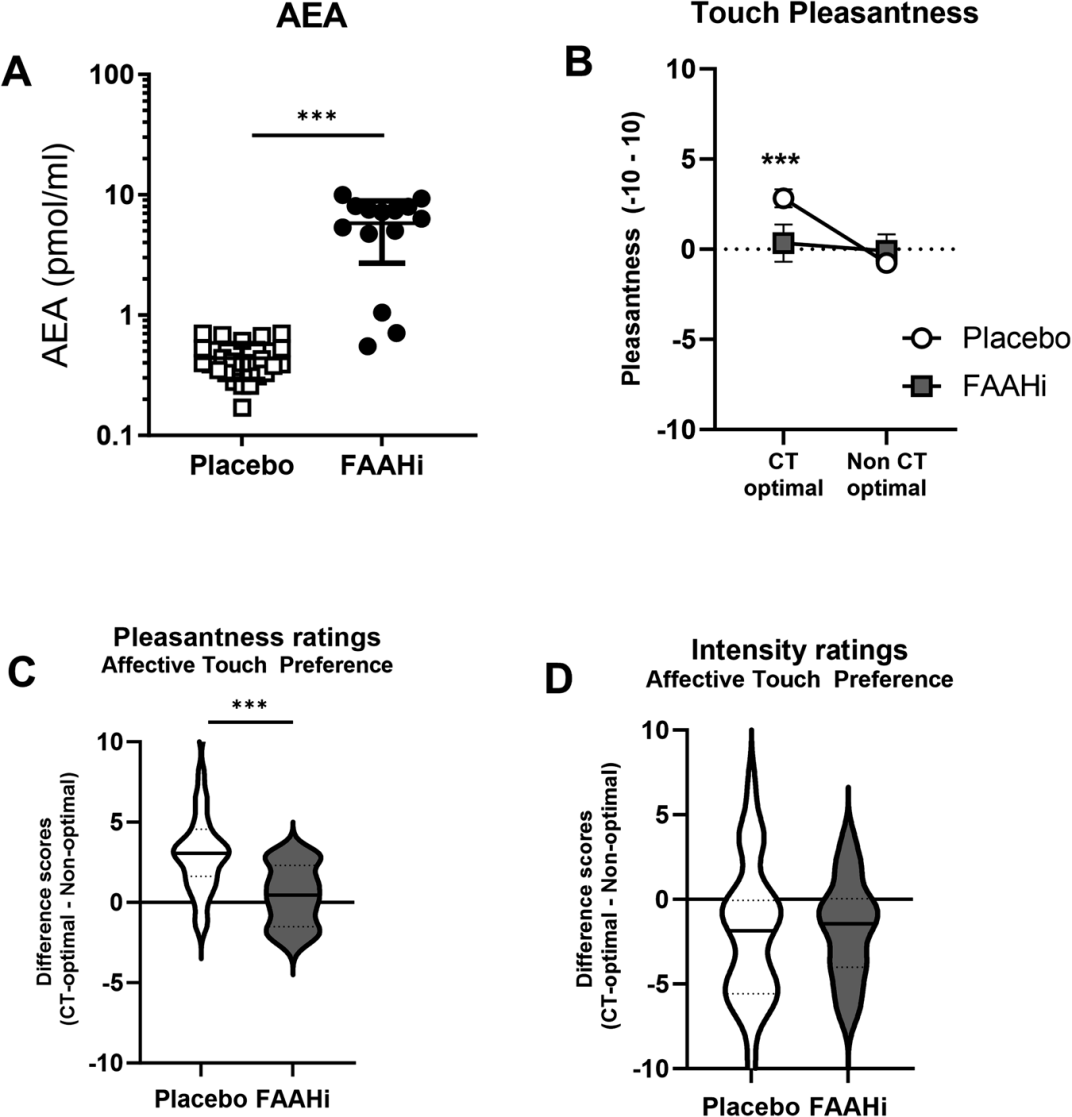
FAAH inhibition is associated with reduced preference for CT-optimal, affective touch. **A** FAAH inhibition significantly elevated basal AEA levels. **B** There is an interaction between treatment (PBO, FAAHi) and touch type. **C** The placebo group shows a significant preference for CT-optimal affective touch; this effect is absent in the FAAHi group. **D** There is no group difference in ratings of touch intensity. ***p < 0.002.

As expected, AEA levels were significantly higher in the FAAHi group compared to PBO (F(1,38) = 82.3, p < 0.001, partial η^2^ = 0.68; Fig. 3A). There were no group differences in peripheral levels of 2-AG (p = 0.14), Cortisol (p = 0.40), or Oxytocin (p = 0.76).

#### Observed touch task

##### Pleasantness ratings

There was a main effect of Touch Type (F(1,42) = 17.0, p < 0.001, partial η^2^ = 0.29; Fig. 3B) and a Touch Type × Treatment interaction (F(1,42) = 10.5, p = 0.002, partial η^2^ = 0.20), but no main effect of Treatment (p = 0.29). Post-hoc follow-up tests revealed that CT-optimal touch was rated as more Pleasant in the PBO group (t(27) = 5.25, p < 0.001; Fig. 2C), as would be expected. However, there was no preference for CT-optimal touch in the FAAHi group (p = 0.19).

##### Intensity ratings

There was a main effect of Touch Type (F(1,42) = 10.8, p = 0.002; Fig. 3D), such that CT-optimal touch was rated as less intense than non-optimal (t(43) = 3.56, p < 0.001). There was no effect of treatment (p = 0.93), nor was there a treatment x Touch Type interaction (p = 0.68).

#### Affective Image Task

##### Valence ratings

**Fig. 4:**
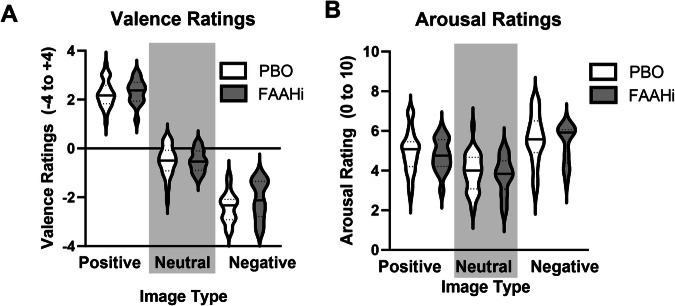
FAAH inhibition does not impact responses to affective images. Across all individuals, Positive images were rating more positively and Negative images were rated more negatively (**A**). Positive and Negative images were rated as more arousing than Neutral images (**B**). There was no effect of FAAH inhibition on ratings of Valence or Arousal.

There was a main effect of stimulus type (F(2,86) = 613, p < 0.001, partial η^2^ = 0.93; Fig. 4A), but no effect of treatment (p = 0.26) or treatment × stimulus interaction (0.47). Post hoc tests revealed that Positive images were rated more positively than Neutral (t(44) = 22.9, p < 0.001) and Negative images (t(44) = 18.3, p < 0.001), and Negative images were rated more negatively than Neural (t(44) = 18.3, p < 0.001).

##### Arousal ratings

There was a main effect of stimulus type (F(2,86) = 63.3 p < 0.001, partial η^2^ = 0.60; Fig. 4B) such that Positive (t(44) = 8.09, p < 0.001) and Negative (t(44) = 12.2, p < 0.001) images were rated as more arousing than Neutral images. There was no effect of Treatment (p = 0.49) or Treatment × Stimulus interaction (p = 0.94).

## Discussion

The eCB system is a critical modulator of stress responses [15] and is also believed to play a role in balancing social approach and avoidance behaviors [16]. Accordingly, the eCB system is proposed as a novel therapeutic target in disorders characterized by impairments in social processing, including social anxiety [17] and autism spectrum disorder [18]. However, evidence linking the eCB system function to social behavior in humans is limited. Here, we explored the role of the eCB system, and AEA in particular, in the perception of socially relevant, CT-optimal touch. In a heterogenous clinical population, we find that *greater* AEA is related to *less* preference for affective, CT-optimal touch. We confirmed the direction of this relationship in a subsequent study in which healthy adults received a pharmacological intervention that elevated AEA via FAAH inhibition. Again, *elevated* AEA was associated with *reduced* preference for CT-optimal touch. Across studies, the effect of elevated AEA was specific to social processing, as there was no impact of AEA on processing of emotional images.

Our results are contrary to the idea that AEA may facilitate prosocial behavior, as we find that *more* AEA is related to *less* preference for socially relevant, CT-optimal touch. However, most preclinical literature has explored the effects of elevated AEA via FAAH inhibition in a “deficit state”—that is, in animal models of chronic stress or following induction of specific ASD-like traits [24–28]. The few studies that have administered FAAH inhibitors under naïve conditions corroborate our findings [29, 30]. Similarly, most human studies have investigated AEA function in clinical populations with dysregulated social processing [17, 18, 31], but data exploring AEA function in relation to social processing in non-clinical human populations are largely absent.

Interestingly, we did not find an effect of FAAH inhibition, and subsequent increases in AEA, on oxytocin levels. This is in contrast to preclinical findings showing that AEA may mediate the pro-social effects of oxytocin [32]. However, we have previously shown that context can significantly impact touch-related dynamic increases in oxytocin [13]. Thus, a more temporally controlled assessment of dynamic AEA and oxytocin changes, particular in relation to observed versus experienced touch, could provide more insight into this potential association in humans.

One possible interpretation of our unexpected results is that we hit a “ceiling effect” on the stress mitigating effects of AEA and touch, as both are proposed as potential stress buffers. Humans with elevated AEA via genetics [19] or pharmacology [20] are protected against stress-induced increases in negative affect. Pharmacological elevation of AEA specifically has been shown to attenuate subjective and autonomic stress reactivity [20]. Affective touch also attenuates psychophysiological stress reactivity [11, 12, 33]. Thus, AEA and affective touch may have similar stress buffering capabilities. As a result, in non-stress conditions, there may be a ceiling effect on the positive attributes of CT-optimal touch, as we find here. What remains to be determined is how this relationship changes in the context of acute or chronic stress, where AEA and touch may each have more pronounced affective impact.

This state-dependent effect on social behavior has been described in relation to the opioid system [34]. For instance, motivation for social touch is increased following stress exposure, and for those pretreated with an opioid agonist, subjective wanting of touch is further facilitated [12]. However, neither opioid stimulation nor blocked impacts touch perception or wanting in the absence of stress [35]. Interestingly, while *negative* states can drive increased wanting and hedonic responses to touch, *appetitive* states do not necessarily impact touch processing [33]. In contrast, other pharmacological interventions, including MDMA, can increase the perceived pleasantness of affective, CT-optimal touch in the absence of stress [36]. MDMA is also proposed as an intervention for psychiatric indications characterized by dysregulation of social processing, such as ASD [37]. Thus, it remains to be seen how these different interventions— MDMA and elevated AEA—could promote therapeutic outcomes in ASD populations, particularly in relation to social processing.

Our findings are limited by the cross-sectional and between-subjects design of our studies, precluding comparison in how dynamic changes in AEA may impact touch perception, particularly in stressful contexts. Moreover, the touch task employed herein is advantageous due to the controlled nature of the stimuli but may not be representative of naturalistic touch encounters [38]. Furthermore, other social processing modalities were not addressed, limiting the generalizability of our findings. Lastly, the sample size in Study 2 was small, requiring replication of these effects in larger cohorts. However, our results provide clear next steps in exploring the role of the eCB system, and AEA in particular, in social processing and adds much needed human data in this domain.

## Supplementary information

Supplemental Materials
