# Supplementary material for: Endocannabinoid contributions to the perception of socially relevant, affective touch in humans
Source: Neuropsychopharmacology. 2025 Jan 22;50(5):849–55. doi: 10.1038/s41386-025-02053-y (PMC11914470; doi:10.1038/s41386-025-02053-y)
Supplement: Supplementary file 1 — Supplemental Materials [file 41386_2025_2053_MOESM1_ESM.docx]

Jones, Haggarty, et al., **Endocannabinoid contributions to the perception of socially relevant, affective touch in humans: Evidence from clinical populations and a randomized controlled trial**

Supplemental Information

**Study 1**

**Materials and Methods**

**Participants**

A first screening of medical records verified prospectively documented childhood maltreatment (CM) exposure, presence or absence of lifetime SUD diagnosis or contact with SUD clinics. Those who had no CM, had emigrated, deceased, lacked contact details, had current or life-time schizophrenia, bipolar or psychotic disorder, organic brain disorder, current suicidality, or cognitive impairment were excluded at this first selection. Medication use was assessed with self-reports and/or medical records. Participants prescribed psychotropic medications were included provided stable use for at least three months (Table 1). Participants prescribed central stimulants or melatonin omitted medications during the day and the evening before the experimental sessions. A study flow-chart is presented in Figure S1.

**Screening session**

During this session, participants underwent a psychiatric clinical assessment by a trained research nurse or study physician, and a structured Mini International Neuropsychiatric Interview (MINI-7),^1^ for DSM-5,^2^ Swedish version.^3^ Lifetime SUD was identified using the regional health care register and contact with addiction clinics, and current SUD was assessed using the MINI, self-reported current problems and urine drug screens. Controls were at this point excluded if CM was identified in their medical records, during clinical assessment or with the MINI-7 post-traumatic stress disorder (PTSD) module. After determining eligibility, participants received research information, provided written informed consent, and completed self-report questionnaires. Questionnaires assessed self-reported CM, using the Childhood Trauma Questionnaire-Short Form (CTQ).^4,5^ Current alcohol and drug use severity were assessed using the Alcohol Use Disorders Identification Test (AUDIT^6^) and the Drug Use Disorders Identification Test (DUDIT^7^). Emotion regulation and personality traits were assessed using the Difficulties in Emotion Regulation Scale (DERS-16)^8^ and NEO Five-Factor Inventory (NEO-FFI-3)^9^ questionnaires.

**Questionnaires**

The 28-item version, validated in Sweden^10^ of the Childhood Trauma Questionnaire-Short Form (CTQ), was used, with 25 clinical items equally distributed across five subscales including emotional abuse, emotional neglect, sexual abuse, physical neglect and physical abuse.^4,5^ Items are rated 1 (never true) to 5 (very often true), generating a total CTQ-score between 25 and 125, and subscale scores ranging between 5 and 25. Three additional items assess minimization/denial. The AUDIT^6^ is a well-established, 10–item questionnaire measuring alcohol use problems (total score range 0–40). Scores between 0–7 indicate low-risk alcohol use, whereas scores of 8+ indicate hazardous drinking. The DUDIT^7^ is a 11-item questionnaire measuring drug use problems (total score range 0–44). For males, scores of 6+, and for females, scores of 2+ indicate problematic drug use. The DERS-16 is a self-report questionnaire (short form) that addressed the ability to identify and regulate emotions. The CPRS^8,12^ is a 19-item self-report version covering depression, anxiety and obsessional symptoms. The CPRS comprises the Montgomery-Asberg Depression Rating Scale and the Brief Anxiety Scale, which are both subscales drawn from the CPRS plus two questions about obsessional symptoms.

**Behavioral Session**

All participants completed the following tasks: the Vicarious Touch task (*reported here*), a Fear Conditioning and Extinction Task, an Affective Image task (*reported here*), a standardized stress task, and a second Affective Image task. Other tasks are reported in (Perini*, Mayo* et al., 2023 *Molecular Psychiatry*).

### **Endocannabinoid analysis**

The lipid concentrations were analysed using a liquid chromatography tandem mass spectrometry (LC-MS/MS) method based on a previously published method.^13^ Before the measurements, lipids were extracted from plasma following a previously described protocol.^14^ All standards (Arachidonylethanolamide, AEA; 2-arachidonoylglycerol, 2-AG; Palmitoylethanolamide, PEA; Oleoyl ethanolamide, OEA), and internal standards (Arachidonylethanolamide-d4, AEA-d4; 2-arachidonoylglycerol-d5, 2-AG-d5; Palmitoylethanolamide-d4, PEA-d4; Oleoyl ethanolamide-d4, OEA-d4) were purchased from Cayman Chemicals (Ann Arbor, MI, USA). Briefly, plasma samples were vortexed after being thawed on ice and 1.2 mL 100% acetonitrile (ACN; Sigma Aldrich, USA) was added to 300 µL of the sample and vortexed. 30 µL of deuterated internal standard [AEA-d4, OEA-d4, PEA-d4 (50 nM)) and 2AG-d5 (1000nM); Biotage, Sweden] was added to each plasma and blank sample, before centrifugation (5 min, 3000g, 4^o^ C). The supernatant was transported to 4.5 mL MilliQ-H_2_O with 0.133% triflouro acetic acid (TFA; Sigma Aldrich, USA). Thereafter the samples were transferred to C8 Octyl SPE columns (6 mL, 200mg; Biotage, Sweden). Prior to transferring the samples, the C8 Octyl SPE columns were activated with 1ml Methanol (Merck, Darmstadt, Germany) and washed with 1mL MilliQ-H_2_O using a Biotage ® Pressure+ 48 machine. After the samples were added, the columns were washed with ACN (20% with 0.1% TFA) and samples were eluted with ACN (80% with 0.1% TFA). The eluates were evaporated to dryness in a SpeedVacc (Thermo Fisher, Ann Arbor, MI, USA) and stored in -80 °C until analysis. On the day of the analysis, the samples were reconstituted in 30µl Mobile phase A (methanol-milliQ water-acetonitrile (4/4/2) (v/v/v) with 0.1 % (v/v) formic acid and 1g/L ammonium acetate), then vortexed and transferred into glass vials designed for the LC-MS/MS. The injection volume was 10 μL. We used an LC-MS/MS system consisting of a Thermo Scientific Accela AS auto sampler and Accela 1250 pump coupled to a Thermo Scientific TSQ Quantum Access max triple quadrupole mass spectrometer with a HESI II probe as ionization source. LC was performed using gradient elution with mobile phase A, and mobile phase B (containing methanol-ACN (7/3) (v/v) with 0.1% (v/v) formic acid and 1g/L ammonium acetate). The gradient elution was applied with a constant flow of 250 μL/min. We started with 100% mobile phase A during the first 1.5 min and followed this using a linear increase towards 100% mobile phase B, which was achieved after 9 min in total. Between the 11th and 12th min the gradient changed linear to 100% mobile phase A, which was maintained for 1 min. An Xbridge C8 analytical column (2.1 mm × 150 mm) with the particle size 2.5 µm obtained from Waters (Dublin, Ireland) was used. We used the following selected reaction monitoring (SRM) (m/z) transitions: 348.3/ 62.4; 326.3/62.4; 300.3/62.4; and 379.3/287.3 for AEA, OEA, PEA, and 2-AG, respectively. For the corresponding internal standards, we used the following transitions: 352.3/ 62.4; 330.3/62.4; 304.3/62.4; and 384.3/287.3 for AEA-d4, OEA-d4, PEA-d4, and 2-AG-d5, respectively. The linearity of the measuring ranges was assessed with standard curves ranging from 1-25 nM for AEA and 10-500 nM for OEA, and PEA, and 50-1250 nM for 2-AG in duplicate. The linearity of the standard curves was R^2^ ≥ 0.9 for all analytes. Isotopic dilution was used for quantification of the analytes, performed according to their area ratio of their corresponding deuterated internal standard signal area. Linear regression and X^2^ weighting were applied. Undetected levels were considered as 0 nM. Xcalibur® (version 2.1, Thermo Scientific) software was used for peak integration and quantification.

**FIGURES**

**Figure S1.** CONSORT flow-chart of study participants. ^1^ Emotional Conflict Task; ^2^ Negative Affect Picture Task; ^3^ Resting State.

**References**

1. Sheehan DV, Lecrubier Y, Sheehan KH, et al. The Mini-International Neuropsychiatric Interview (M.I.N.I.): the development and validation of a structured diagnostic psychiatric interview for DSM-IV and ICD-10. *The Journal of clinical psychiatry*. 1998;59 Suppl 20:22-33;quiz 34-57.

2. American Psychiatric Association. *Diagnostic and Statistical Manual of Mental Disorders: DSM-5*. 5th ed. American Psychiatric Association; 2013.

3. Allgulander C, Nilsson B. Rikstäckande primärvårdsstudie: Var fjärde patient lider av ångest och depression. *Lakartidningen*. 2003;100:832-8.

4. Bernstein DP, Fink L, Handelsman L, et al. Initial reliability and validity of a new retrospective measure of child abuse and neglect. *Am J Psychiatry*. Aug 1994;151(8):1132-6. doi:10.1176/ajp.151.8.1132

5. Bernstein DP, Stein JA, Newcomb MD, et al. Development and validation of a brief screening version of the Childhood Trauma Questionnaire. *Child Abuse Negl*. Feb 2003;27(2):169-90. doi:10.1016/s0145-2134(02)00541-0

6. World Health O. AUDIT: the Alcohol Use Disorders Identification Test : guidelines for use in primary health care / Thomas F. Babor ... [et al.]. 2nd ed ed. Geneva: World Health Organization; 2001.

7. Berman AH, Bergman H, Palmstierna T, Schlyter F. Evaluation of the Drug Use Disorders Identification Test (DUDIT) in criminal justice and detoxification settings and in a Swedish population sample. *Eur Addict Res*. 2005;11(1):22-31. doi:10.1159/000081413

8. Bjureberg J, Ljótsson B, Tull MT, et al. Development and Validation of a Brief Version of the Difficulties in Emotion Regulation Scale: The DERS-16. *J Psychopathol Behav Assess*. Jun 2016;38(2):284-296. doi:10.1007/s10862-015-9514-x

9. Costa PT, McCrae RR. *Revised NEO personality inventory (NEO PI-R) and NEP five-factor inventory (NEO-FFI) : professional manual*. Psychological Assessment Resources; 1992:vi, 101 p.

10. Gerdner A, Allgulander C. Psychometric properties of the Swedish version of the Childhood Trauma Questionnaire—Short Form (CTQ-SF). *Nordic Journal of Psychiatry*. 2009/01/01 2009;63(2):160-170. doi:10.1080/08039480802514366

11. Kallmen H, Wennberg P, Bergman H. Psychometric properties and norm data of the Swedish version of the NEO-PI-R. *Nord J Psychiatry*. Oct 2011;65(5):311-4. doi:10.3109/08039488.2010.545433

12. Svanborg P, Asberg M. A new self-rating scale for depression and anxiety states based on the Comprehensive Psychopathological Rating Scale. *Acta psychiatrica Scandinavica*. Jan 1994;89(1):21-8.

13. Stensson N, Ghafouri N, Träff H, Anderson CD, Gerdle B, Ghafouri B. Identification of lipid mediators in peripheral human tissues using an integrative in vivo microdialysis approach. *Journal of Analytical and Bioanalytical Techniques*. 2016;7:306doi:10.4172/2155-9872.1000306

14. Stensson N, Ghafouri B, Gerdle B, Ghafouri N. Alterations of anti-inflammatory lipids in plasma from women with chronic widespread pain - a case control study. *Lipids Health Dis*. Jun 12 2017;16(1):112. doi:10.1186/s12944-017-0505-7

**Study 2**

**Supplemental Methods and Materials**

**
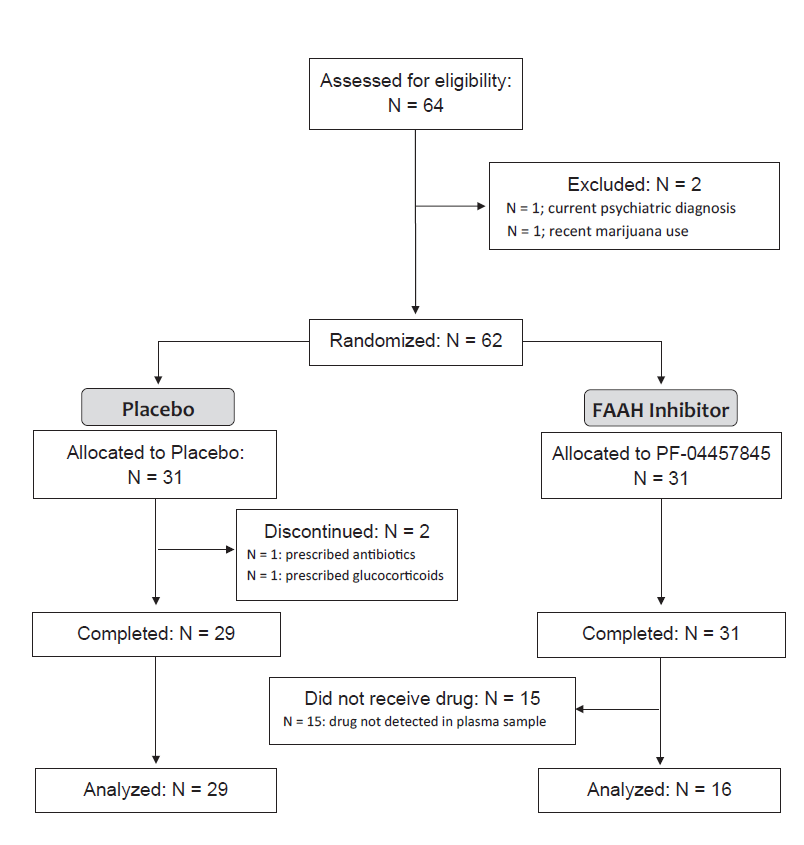
**

**Figure S2:** Study flowchart. As a result of a pharmacy error, a number of participants randomized to FAAH inhibition PF-04457845 (*n* = 15) did not receive active medication (see below for more information). Results presented are from per-protocol analysis of participants to biochemically confirmed PF-04457845 exposure.

| Visit | Screening | Visit 1 | Visit 2 | Follow-up phone call |
| --- | --- | --- | --- | --- |
| **Time point** | Max. 14 days prior FDOT^a^ | 9 days (± 0) post FDOT^a^ | 10 days (± 0) post FDOT^a^ | 24 days (± 3 days) post FDOT^a^ |
| **Evaluation of inclusion/exclusion criteria** | X |  |  |  |
| **Pregnancy test^b^** | X |  |  |  |
| **Safety measures^c^** | X | X | X |  |
| **Safety blood samples^d^** | X |  | X |  |
| **Informed consent** | X |  |  |  |
| **Blood sample, genotyping** | X |  |  |  |
| **Urine sample, illicit drug screen^f^** | X |  |  |  |
| **Randomization** | X |  |  |  |
| **Distribution of study drug/placebo** | X |  |  |  |
| **Self-report questionnaires** (NEO-FFI, STAI-T) | X |  |  |  |
| **Behavioral tasks** |  | X | X |  |
| **Psychophysiological measurements^e^** |  | X | X |  |
| **Self-report questionnaires** (STAT-S, PANAS, POMS) |  | X | X |  |
| **Blood samples** (endocannabinoids, cortisol) |  | X | X |  |
| **Adverse event registration** |  | X | X | X |
| **Compliance control** |  | X | X |  |
| **Collection of study drug/placebo** |  |  | X |  |
| **Supplemental Table 1: Data collection procedures**. ^a^FDOT: First day of treatment, ^b^Only for women of child-bearing potential, ^c^blood pressure, heart rate, well-being and psychiatric symptoms, ^d^AST, ALT, GGT, WBC with diff., Hb, MCV, TPK, LPK and CRP, ^e^, heart rate, respiration, skin conductance, facial EMG; **^e^**THC, amphetamine, methamphetamine, opioids, bensodiazepines, cocaine, buprenorphin, methadone, tramadol, oxycontin, fentanyl, and clonazepam. | | | | |

***Inclusion and Exclusion criteria***

Participants were required to be 18 years or older and provide informed consent. Exclusion criteria include: lifetime diagnosis of psychosis or bipolar disease, current axis 1 diagnosis; as determined by a history, clinical examination and MINI interview carried out by appropriately trained staff; ongoing (within the last month) psychiatric medication; current (within the last month) use of illicit drugs, as identified using the Drug Use Disorder Identification Test (DUDIT) or a positive urine screen; co-medication with CYP3A inhibitors, CYP3A inducers or P-glycoprotein substrates; any other current medication or medical condition that in the judgment of the investigator could interfere with treatment; pregnancy or nursing. To be eligible, women of childbearing potential (WOCBP) must have a negative serum or urine pregnancy test prior to the start of study drug. WOCBP and males with WOCBP partners must agree to use a method of contraception that is highly effective for the duration of the study and for at least 28 days after the intake of the study drug.

***Drug (PF-04457845)***

PF-04457845 (Pfizer, Groton, VT, USA) is an orally available, highly selective covalent inhibitor of FAAH (1, 2). The pharmacokinetics have been characterized in healthy adult volunteers in single doses (dose range 0.1 to 40 mg) or in multiple doses (dose range 0.5 to 8 mg). Maximal plasma concentrations of the drug were reached within two hours of administration and the half-life is estimated to be 12 to 23 hours. Multiple dosing produces steady-state plasma concentrations by day 7 of dosing. The 4 mg daily dose was selected because it maximally inhibits FAAH and is well tolerated, while higher doses do not produce any detectable advantage. No serious adverse events were reported in any of the completed phase I studies, or clinical studies including patients with osteoarthritis or cannabis use disorder (1–5). Positron emission tomography has shown that a 1 mg dose of PF-04457845 effectively inhibits FAAH (>95%) in the human brain (6). Following multiple dosing of 4mg, the washout period is estimated to take 10 days.

The drug supply used in this study was originally designated for a clinical trial assessing the efficacy of PF-04457845 on co-morbid PTSD and alcohol use disorder (AUD) in women at the Karolinska Institute, Stockholm, Sweden (EudraCT 2014-002456-9). Drug and matched placebo were provided in bulk by Pfizer, to be packaged, labeled and released by a local contractor and the hospital pharmacy. Like all FAAH inhibitor trials, this trial was placed on a clinical hold after a report that the FAAH inhibitor BIA 10-2474 had caused serious toxicity including 1 death in a Phase 1 study (7, 8). These effects were later attributed to off-target toxicity of this specific compound (9), and the clinical hold was lifted. Meanwhile, however, the original study had been dropped, and replaced by a behavioral treatment trial that is currently ongoing (10).

When the clinical hold was lifted, and it was clear that PF-04457845 was safe to use in humans, the remaining drug was therefore re-purposed for the current study with approvals of the Swedish Medical Products Agency and concurrence from Pfizer. The drug was sent from the pharmacy at the Karolinska Hospital in Stockholm to be re-labeled by a contractor (Oriola; Stockholm, Sweden). The re-labeled medication was then sent to the pharmacy at University Hospital in Linköping, Sweden, and distributed to participants as previously detailed. Unfortunately, this involved 1 pharmaceutical company, two contractors, and two different hospital pharmacies.

In the current study, we initially monitored compliance by pill-count, and debriefed all subjects on their compliance during follow-up, which was part of the study to monitor safety. We then analyzed AEA levels as an objective biomarker of target engagement. In these data, approximately half (N = 15) of the participants randomized to the FAAHi group had unaffected AEA levels, while the remaining participants had levels that were on average 10-fold elevated. We then analyzed drug levels, and found that individuals with unaffected AEA levels also did not have detectable drug levels. Of note, we analyzed samples from all subjects (placebo and FAAHi-treated), and could confirm that no participants in the group randomized to placebo had received active drug.

Finally, we carried out an extensive audit, in which each participant was re-contacted and debriefed personally by the sponsor / PI. Participants were informed of the situation and asked whether they had any new information to provide to help account for the findings, with no penalty to themselves. While in some cases admitting to behaviors that could be considered problematic in the study (one case: THC use; two other cases: one or more episodes of binge drinking), they all ensured that their initial report on drug compliance was correct.

In conclusion, all subjects randomized to receive placebo were objectively confirmed to have received their allocated treatment, while only 16 subjects randomized to receive active medication received it, with the remainder of this group inadvertently receiving placebo due a packaging / labeling error.

| **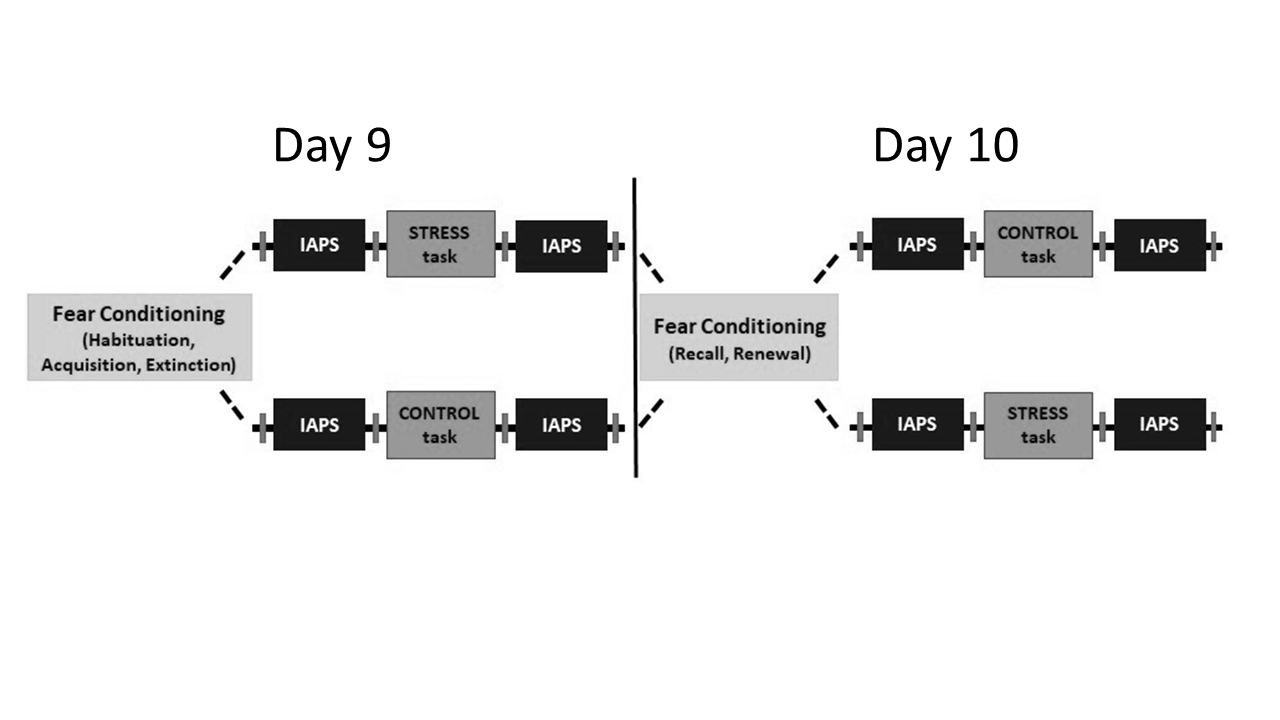** |
| --- |
| **Supplemental Figure S3 Study schematic.** Participants completed laboratory sessions on days 9 and 10 of dosing. All participants first completed fear conditioning tasks, including habituation, acquisition, and extinction. Then they completed an affective image task (IAPS), stress or control, and another affective image task. On day two, they underwent recall of fear extinction and renewal of fear. They again completed an affective image task (IAPS), stress or control (whichever was not completed on day 9), and another affective image task. Blood samples were taken via an indwelling intravenous catheter before and after IAPS and stress/control tasks. Participants were randomized to receive stress or control first. Tasks reported here always included the post-control IAPS and a subsequent Observed Touch Task completed after. |

***Biochemical Analysis***

Endocannabinoids. Assay of plasma levels of the endocannabinoids N-arachidonoylethanolamide (AEA) and 2-arachidonoylglycerol (2-AG) and the fatty acid ethanolamides palmitoylethanolamide (PEA) and oleoylethanolamide (OEA) was performed using mass spectrometry. For each sample, 500ul of plasma was directly pipetted into 2ml of acetonitrile with 5 nmol of d8-2-AG, 5 pmol of d8-AEA, 40 pmol d4-PEA, and 40 pmol d4-OEA. Samples were sonicated for 30min in an ice bath and incubated overnight at -20°C to precipitate proteins. The following day samples were centrifuged at 1500xg to remove particulates. The supernatant from each sample was transferred to a new glass tube and evaporated under nitrogen, the tube was then washed once with 350ul acetonitrile (to recapture any lipids adhering to the glass wall) and the acetonitrile was dried under nitrogen gas again. After completely drying, the samples were re-suspended in 200ul of acetonitrile and stored at -80°C until analysis by liquid chromatography mass spectrometry. Analysis in mass spectrometry was performed exactly as previously described (18).

Detection of PF-04457845. The presence of the FAAH inhibitor PF-04457845 from plasma was performed using mass spectrometry and was based on a previously described method (5). First, 500ul of plasma was injected into 2 ml of acetonitrile and samples were sonicated for 30min in an ice bath and incubated overnight at -20°C to precipitate proteins. The following day samples were centrifuged at 1500xg to remove particulates. The supernatant from each sample was transferred to a new glass tube and evaporated under nitrogen, the tube was then washed once with 350ul acetonitrile (to recapture any lipids adhering to the glass wall) and the acetonitrile was dried under nitrogen gas again. After completely drying, the samples were re-suspended in 100ul of 50:50 methanol:water solution and stored at -80°C until analysis by liquid chromatography mass spectrometry.

The LC-MS/MRM analysis was performed using an Eksigent Micro LC200 coupled with an AB Sciex QTRAP 5500 mass spectrometry (AB Sciex, Ontario, Canada). Chromatographic separation of the analytes was carried out on an Eksigent Halo C18 column (2.7 µm, 0.5 × 50 mm, 90Å, AB Sciex). The mobile phase A was 0.1% formic acid in water and the mobile phase B was 0.1% formic acid in acetonitrile. The gradient program was shown as the following: 0-1.2 min (55% B), 1.2-1.7 min (55-95% B), 1.7-3.0 min (95% B), 3-3.5 min (95-55% B), 3.5-5.0 min (55% B). The flow rate is 30 µL/min and the injection volume was 2.0 µL. The data were acquired in positive electrospray ionization (ESI) and multiple reaction monitoring (MRM) mode. MRM transitions and collision energy (CE) of all compounds were listed in Table 1. Ion spray voltage was 5500 V. Nebulizer gas (GS 1), auxiliary gas (GS 2), curtain gas (CUR) were 30, 30, 35 (arbitrary units). Collision gas was set as Medium. Declustering potential (DP), entrance potential (EP) and cell exit potential (CXP) were 80, 7 and 14 V.

For quantification, a stock solution of PF-04457845 (1.0 mg/mL) was used to prepare a set of calibrators ranging from 0.1 ng/mL to 50 ng/mL (0.1, 0.5, 1.0, 5.0, 10, 50) with methanol/water (50:50, *v/v*) via a serial dilution. Because the isotopic labelled PF internal standard was not available, concentrations of PF in the samples were determined by an external calibration curve composed by the six calibrators. PF levels were then normalized to quantity per ml of plasma. The lower detection limit was 1 ng/ml and so only samples which registered above this range were quantifiable.

**Supplemental Results**

**Supplemental Figure S4: No effect of FAAH inhibition on oxytocin levels.**

|  | ***Placebo***  ***N=29*** | ***Drug (confirmed)***  ***N=16*** | *Drug (total)*  *N=31* | |  |
| --- | --- | --- | --- | --- | --- |
| **Non-serious adverse events during treatment (10 days)** | | |  | |  |
| *Headache* | **5** | **3** | 3 | |  |
| *Cold/respiratory congestion* | **2** | **3** | 8 | |  |
| *Fatigue* | **2** | **1** | 3 | |  |
| *Anxiety* | **2** | **1** | 5 | |  |
| *Sleep difficulties* | **2** |  |  | |  |
| *Improved sleep* |  | **1** | 1 | |  |
| *Nausea* | **4** | **1** | 2 | |  |
| *Cardiovascular* |  | **1** | 4 | |  |
| *Herpes* |  |  | 1 | |  |
| *Joint/muscle pain* | **2** |  | | 1 |  |
| **Incidence of non-serious adverse events** | | | | |  |
| *Number of participants* | **15** | **8** | | 19 |  |
| *Percentage* | **52%** | **50%** | | 61% |  |
|  | | | | |  |
| **Supplemental Table S2: Non-serious adverse events during treatment.** Amount and type of self-reported, non-serious adverse events reported by patients randomized to receive placebo (N = 29) or drug (PF-04457845; N = 31) once a day for ten days. The treatment group is divided into those who were confirmed to receive the drug (confirmed; N = 16), and those who were randomized to the treatment group but did not receive the drug due to a pharmacy error (intended; N = 15; not shown). Incidence of events is calculated as the number of individuals reporting one or more non-serious adverse event throughout the treatment period. No serious adverse events were reported in any group. | | | | | |

**Supplemental References**

1. K. Ahn *et al.*, *J. Pharmacol. Exp. Ther.* **338**, 114–124 (2011).

2. J. P. Huggins, T. S. Smart, S. Langman, L. Taylor, T. Young, *Pain*. **153**, 1837–1846 (2012).

3. D. C. D’Souza *et al.*, *The Lancet Psychiatry*. **6**, 35–45 (2019).

4. D. S. Johnson *et al.*, *ACS Med Chem Lett*. **2**, 91–96 (2011).

5. G. L. Li *et al.*, *British journal of clinical pharmacology*. **73**, 706–716 (2012).

6. I. Boileau *et al.*, *Journal of cerebral blood flow and metabolism : official journal of the International Society of Cerebral Blood Flow and Metabolism*. **35**, 1237–40 (2015).

7. A. Mullard, *Nature Reviews Drug Discovery*. **16**, 447 (2017).

8. A. Kerbrat *et al.*, Acute Neurologic Disorder from an Inhibitor of Fatty Acid Amide Hydrolase. *https://doi.org/10.1056/NEJMoa1604221* (2016), , doi:10.1056/NEJMoa1604221.

9. A. C. M. van Esbroeck *et al.*, *Science*. **356**, 1084–1087 (2017).

10. A. Persson *et al.*, *Journal of addiction medicine*. **11**, 119–125 (2017).
